# Features and Prognosis of Severe Malaria Caused by *Plasmodium falciparum*, *Plasmodium vivax* and Mixed *Plasmodium* Species in Papua New Guinean Children

**DOI:** 10.1371/journal.pone.0029203

**Published:** 2011-12-22

**Authors:** Laurens Manning, Moses Laman, Irwin Law, Cathy Bona, Susan Aipit, David Teine, Jonathan Warrell, Anna Rosanas-Urgell, Enmoore Lin, Benson Kiniboro, John Vince, Ilomo Hwaiwhanje, Harin Karunajeewa, Pascal Michon, Peter Siba, Ivo Mueller, Timothy M. E. Davis

**Affiliations:** 1 School of Medicine and Pharmacology, University of Western Australia, Fremantle Hospital, Fremantle, Western Australia, Australia; 2 Papua New Guinea Institute of Medical Research, Madang, Madang Province, Papua New Guinea; 3 School of Medicine and Health Sciences, University of Papua New Guinea, Boroko, Port Moresby, Papua New Guinea; 4 Department of Pediatrics, Goroka Base Hospital, Goroka, Eastern Highlands Province, Papua New Guinea; Pennsylvania State University College of Medicine, United States of America

## Abstract

**Background:**

Mortality from severe pediatric falciparum malaria appears low in Oceania but *Plasmodium vivax* is increasingly recognized as a cause of complications and death. The features and prognosis of mixed *Plasmodium* species infections are poorly characterized. Detailed prospective studies that include accurate malaria diagnosis and detection of co-morbidities are lacking.

**Methods and Findings:**

We followed 340 Papua New Guinean (PNG) children with PCR-confirmed severe malaria (77.1% *P. falciparum*, 7.9% *P. vivax*, 14.7% *P. falciparum/vivax*) hospitalized over a 3-year period. Bacterial cultures were performed to identify co-incident sepsis. Clinical management was under national guidelines. Of 262 children with severe falciparum malaria, 30.9%, 24.8% and 23.2% had impaired consciousness, severe anemia, and metabolic acidosis/hyperlactatemia, respectively. Two (0.8%) presented with hypoglycemia, seven (2.7%) were discharged with neurologic impairment, and one child died (0.4%). The 27 severe vivax malaria cases presented with similar phenotypic features to the falciparum malaria cases but respiratory distress was five times more common (*P* = 0.001); one child died (3.7%). The 50 children with *P. falciparum/vivax* infections shared phenotypic features of mono-species infections, but were more likely to present in deep coma and had the highest mortality (8.0%; *P* = 0.003 vs falciparum malaria). Overall, bacterial cultures were positive in only two non-fatal cases. 83.6% of the children had alpha-thalassemia trait and seven with coma/impaired consciousness had South Asian ovalocytosis (SAO).

**Conclusions:**

The low mortality from severe falciparum malaria in PNG children may reflect protective genetic factors other than alpha-thalassemia trait/SAO, good nutrition, and/or infrequent co-incident sepsis. Severe vivax malaria had similar features but severe *P. falciparum/vivax* infections were associated with the most severe phenotype and worst prognosis.

## Introduction

The features and prognosis of severe pediatric falciparum malaria have been characterized in observational and intervention studies from sub-Saharan Africa [1]–[4]. Equivalent studies in other epidemiologic contexts are fewer but important differences have emerged. There is some evidence that mortality from severe *Plasmodium falciparum* infections in children is relatively low in the Oceania region [5]–[7], perhaps due to the acquisition of cross-species functional immunity from greater exposure to *P. vivax* in early childhood. Nevertheless, *P. vivax* is itself increasingly recognized as a cause of both complications similar to those seen in falciparum malaria and death [8]. In the case of mixed *Plasmodium* species infections (commonly *P. falciparum/vivax*), there are reports of reduced [9], [10], equivalent [11], [12] and increased [12] morbidity relative to *P. falciparum* mono-infections.

The incidence of complications and death from severe malaria will depend on selection/recruitment strategies, sensitivity of malaria diagnosis/speciation, definitions of severity, inpatient management including identification of co-incident disease, and social, cultural and genetic factors that can influence presentation and clinical course. Apparent inconsistencies between published studies may reflect differences in one or more such factors. The characteristics and outcome of severe malaria are, therefore, best assessed prospectively, in a representative, ethnically homogeneous sample, and with clinical and laboratory data sufficient both to allow accurate diagnosis [13] and the detection of important co-morbidities such as sepsis [14].

We have carried out such a study in children in coastal Papua New Guinea (PNG) presenting with severe malarial illness, a setting in which a high prevalence of alpha-thalassemia and South Asian ovalocytosis (SAO) implies that malaria has had a strong selective effect [15]–[17]. We hypothesized that i) severe falciparum malaria has a low mortality relative to African studies as found previously in simple observational studies [5]–[7], [18], and that ii) severe *P. vivax* and mixed *P. falciparum/vivax* malaria, although less common than that caused by *P. falciparum*, have a similar spectrum of clinical disease and prognosis.

## Methods

### Study sites and local malaria epidemiology

The present study was conducted in Madang and Sepik Provinces on the northern PNG coast where most of the population are subsistence farmers and their families. The annual entomological inoculation rate (EIR) for Madang Province has recently been estimated at 37 for *P. falciparum* and 24 for *P. vivax* [19]. At the Sepik study site, the EIR is 35 for *P. falciparum* and 12 for *P. vivax* [20]. In healthy, asymptomatic Madang children aged 1–10 years, the spleen rate is 13% and the prevalence of parasitemia by microscopy is 8.2% for *P. falciparum* (median [interquartile range (IQR)] 1360 [453–2881]/µL) and 14.1% (348 [226–727]/µL) for *P. vivax* [21]. Approximately 90% of local children have alpha-thalassemia trait [15]. The national human immunodeficiency virus (HIV) seroprevalence is 0.9% [22].

### Ethics statement

The present study was approved by the PNG Institute of Medical Research Institutional Review Board and the Medical Research Advisory Committee of the PNG Health Department, and conducted according to the principles of the Declaration of Helsinki. Written informed consent was obtained from parent(s)/guardian(s) before recruitment.

### Patients

Between October 2006 and December 2009, all children aged 0.5–10 years admitted to Modilon Hospital, the provincial hospital to which the majority of children with severe illness are referred, were assessed for recruitment to an observational study of severe pediatric illness. Inclusion criteria included any of: i) impaired consciousness/coma (Blantyre Coma Score (BCS)<5 [23]), ii) prostration (inability to sit/stand unaided), iii) multiple seizures, iv) hyperlactatemia (blood lactate>5 mmol/L), v) severe anemia (hemoglobin<50 g/L), vi) dark urine, vii) hypoglycemia (blood glucose≤2.2 mmol/L), viii) jaundice, ix) respiratory distress, x) persistent vomiting, xi) abnormal bleeding or xii) signs of shock. These criteria reflect the World Health Organization (WHO) definition of severe malarial illness [13]. All severely-ill blood slide-positive children were considered for recruitment but only those in whom *Plasmodium* species were detected by nested polymerase chain reaction (nPCR) were included in the present analyses. The final speciation as a mono- or mixed infection was based on the nPCR result.

### Clinical assessment and management

After recruitment, a standardized case report form that recorded demographic and medical data was completed [24]. This included details of immunizations, past medical history and recent treatment with antimalarial drugs and antibiotics, as documented in each child's hand-held medical record book. Trained study nurses carried out clinical assessments on admission and an additional detailed neurological examination was performed by study clinicians (LM, ML) on all children admitted with a BCS≤4, regardless of the time of admission. Weekly bedside teaching by the study clinicians was conducted to ensure consistency of clinical assessment by study nurses.

A BCS≤2 was considered deep coma and a BCS≤4 as impaired consciousness at 0.5, 1 or 6 hours after correction of hypoglycemia, a seizure or parenteral anticonvulsant therapy, respectively. Respiratory distress was considered present if the child had i) deep breathing, ii) inter-costal in-drawing, iii) sub-costal recession, iv) persistent alar flaring, v) tracheal tug, and/or vi) respiratory rate>60/minute. Due to variable availability and safety concerns, chest radiography was performed in a minority of children with respiratory symptoms. Inpatient management, including the decision to perform lumbar puncture (LP), was co-ordinated by attending ward clinicians under PNG national guidelines [25] and included intravenous dextrose/saline and antibiotics (chloramphenicol 25 mg/kg by intramuscular injection 6-hourly), intramuscular artemether (3.2 mg/kg on admission and 1.6 mg/kg daily until oral antimalarial therapy could be tolerated), and blood transfusion if required (in the absence of cardiovascular compromise, at a hemoglobin<40 g/L). Each child was reviewed at least daily until discharge/death. At discharge and, where possible, at the child's home two months post-discharge, the presence of residual neurologic deficits was assessed.

### Laboratory Procedures

A baseline venous blood sample was taken for microscopy and rapid diagnostic testing (RDT) (ICT Diagnostics, Brookvale, Australia). Giemsa-stained thick blood smears were examined and the parasitemia quantified independently by two skilled microscopists with discrepancies adjudicated by a third microscopist [26]. Parasite densities were calculated from the number of parasites per 200 white cells and an assumed total peripheral white cell count of 8,000/µL. The final density was the geometric mean of the two values [26]. Additional on-site tests comprised whole blood glucose (Hemocue, Ängelholm, Sweden) and lactate (Lactate Pro, Arkray, Japan), and a full blood count (Coulter Ac·T diff, Beckman Coulter, Brea, USA).

When sufficient blood was available from the admission venepuncture after the above tests, 1–3 mL was placed in Bactec™ Peds Plus™/F bottles (Becton Dickinson, Franklin, USA) and incubated using an automated Bactec™ system. Cerebrospinal fluid (CSF) obtained at LP was examined macroscopically and microscopically [24], and semi-quantitative glucose and protein concentrations obtained by urine dipstick (Acon® Laboratories, San Diego, USA) [27]. If>10 white blood cells/mm^3^ were present, CSF was incubated on chocolate agar for 3 days. Bacterial isolates from blood/CSF cultures were identified using standard procedures [24]. Coagulase-negative staphylococci, *Corynebacterium* and *Bacillus spp.* were considered contaminants. The remaining baseline blood sample was centrifuged promptly and separated plasma and erythrocytes stored at −70°C and −20°C, respectively.

Additional laboratory tests not necessary for initial clinical management were performed subsequently and included i) plasma electrolytes, urea, creatinine, alanine aminotransferase (ALT), total bilirubin, C-reactive protein (CRP) and creatine kinase (COBAS INTEGRA 800, Roche Diagnostics, Mannheim, Germany), ii) *Plasmodium* speciation by nPCR [28] after parasite DNA extraction (QIAamp 96 DNA Blood Mini Kit, QIAGEN, Valencia, CA) and iii) genetic testing for alpha-thalassemia (3.7 kb and 4.2 kb deletions) and SAO (SLC4A1Δ27) mutations after host DNA extraction [29], [30]. Serologic testing for HIV was not routinely performed.

Reference intervals for laboratory tests were determined from 327 healthy Melanesian children who were age- and sex-matched to the present children [21]. Metabolic acidosis was defined as a plasma bicarbonate<12.2 mmol/L, hyperbilirubinemia as a plasma bilirubin>35 µmol/L, an acute inflammatory response as a plasma CRP>64 mg/L (twice the upper limit of the reference interval), and significant liver inflammation as a plasma ALT>90 IU/mL. Schwartz's formula was used to estimate creatinine clearance (CrCl) [31], with impaired renal function defined as a CrCl<75 mL/min.

### Prospective population-based study

To compare the proportions of the present hospitalized cases by *Plasmodium* species with those of community-acquired malaria in a similar epidemiologic situation, we used data from 264 children aged 1–4 years from the Ilaita area of neighbouring East Sepik Province who were followed with fortnightly active morbidity assessment and passive case detection at a local health center between April 2006 and August 2007 [32]. At each visit, an RDT was performed to guide treatment, and blood slides and erythrocytes were retained for diagnostic confirmation by microscopy and PCR, respectively. When a child presented with severe illness, a detailed adverse event report was completed. A consensus determination of incident cases of WHO-defined severe malaria [13] was performed by study clinicians (LM, TMED). The denominator for severe malaria incidence was the total at-risk exposure, regardless of antimalarial therapy.

### Data analysis

Summary data are presented as median and [IQR]. Comparisons of variables between groups were by parametric or non-parametric tests as appropriate, with *post hoc* two-group comparisons by Dunn's test for continuous and Bonferroni's correction for categorical variables. Crude odds ratios (ORs) for the observed frequencies of overlapping clinical phenotypes were calculated using 2×2 contingency tables. Multivariate analysis was performed using backward stepwise logistic regression. Variables other than age were entered based on biological plausibility and *P*<0.20 on univariate regression analysis and the most parsimonious model chosen using Aikake's Information Criterion. A two-tailed significance level of *P*<0.05 was used throughout.

## Results

**Figure 1:**
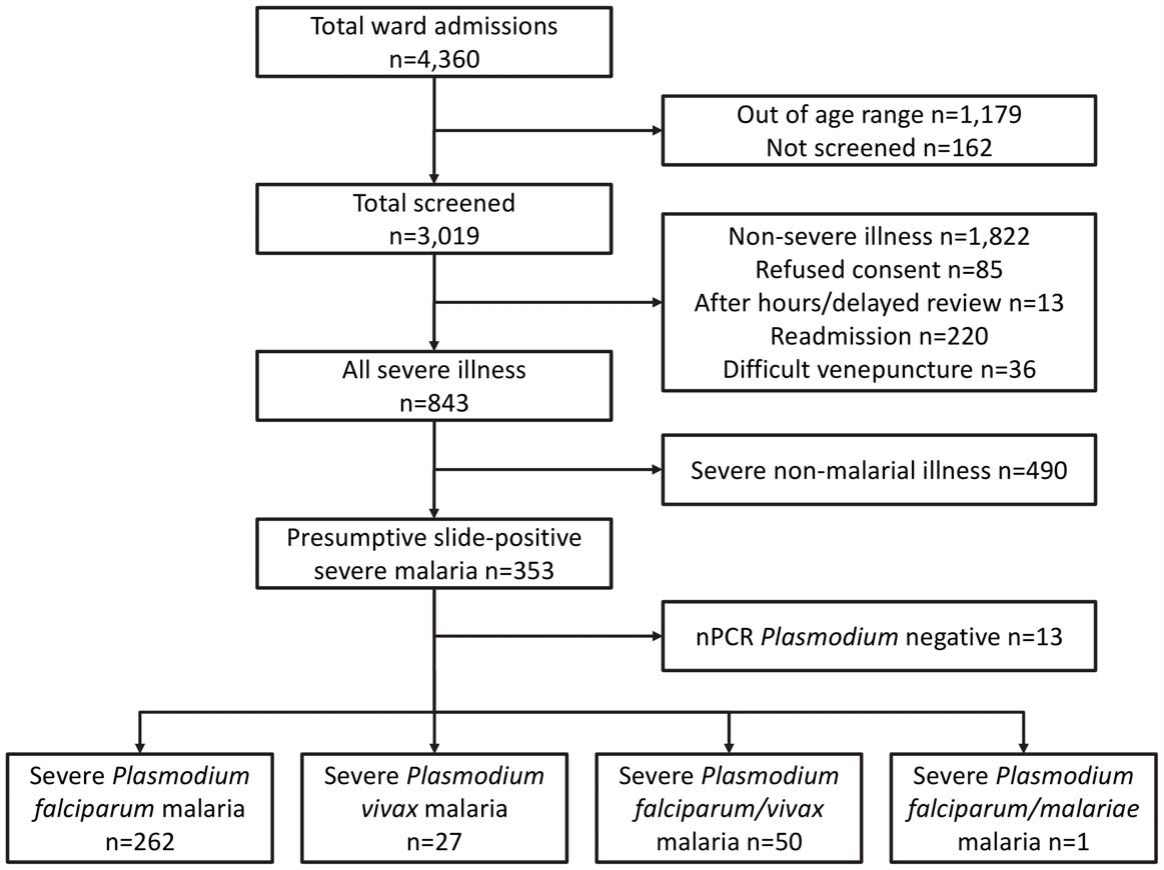
Consort diagram outlining categorization of children presenting to Modilon Hospital, Madang Province, during the study period. Severe malaria cases were identified by clinical and laboratory features at presentation including blood film microscopy, and subsequent nPCR for *Plasmodium* speciation.

During the study, 3,019 of 3,181(94.9%) hospitalized children aged between 6 months and 10 years were screened for inclusion to the study (see Figure 1). Of 353 with presumptive severe malaria based on microscopy, 13 were excluded because *Plasmodium* DNA was undetectable by nPCR. The remaining 340 children had a median age of 40 [29–55] months and 189 (55.6%) were males. In most cases (87.1%), both parents were from Madang or East Sepik provinces, with the rest from other parts of PNG. Based on clinical features, blood film microscopy and nPCR, there were 262 severely-ill children with *P. falciparum* (77.1%), 27 with *P. vivax* (7.9%), 50 with mixed *P. falciparum/vivax* (14.7%) and one with mixed *P. falciparum/malariae* (0.3%; see Table 1).

**Table 1:** Baseline clinical and laboratory data for children with severe malaria categorized by *Plasmodium* species.

	*Plasmodium falciparum* (n = 262)	*Plasmodium vivax* (n = 27)	*Plasmodium falciparum/vivax* (n = 50)	*P*-value*
Age (months)	42 [30–57]	33 [26–61]	36 [29–48]	0.25
Male sex (%)	58.5	55.6	42.0	0.17
Axillary temperature (°C)	37.9 [37.2–38.7]	37.6 [36.9–38.8]	38.0 [37.0–38.4]	0.28
Pulse rate (/minute)	132 [117–145]	133 [119–146]	139 [121–149]	0.37
Respiratory rate (/minute)	32 [28–38]^a^	36 [31.5–43]	38 [31–48]	0.014
Respiratory distress (%)	9.9^b^	29.6	22.0	0.002
Oxygen saturation (%)	98 [97–99]	98 [96.5–98.5]	99 [97–100]	0.049
Spleen size (cm)	2 [0–4]	0 [0–2]^c^	3 [0–5.3]	0.021
Blantyre Coma Score	5 [4–5]^a^	5 [4–5]	4.5 [3–5]	0.018
Impaired consciousness (3–4; %)	20.6	22.2	28.0	<0.0001
Deep coma (≤2; %)	10.3	3.7	22.0	
Multiple/complex fits (%)	21.8	25.9	22.0	0.88
Hemoglobin (g/L)	77 [50–94]^b^	95 [77–105]^c^	73 [50–92]	0.015
Severe anemia (<50 g/L, %)	24.9	11.1	24.0	0.28
Leukocyte count (×10^9^/L)	8.4 [6.0–12.5]	9 [6.6–12.8]	9.0 [7.1–12.3]	0.67
Platelet count (×10^9^/L)	101 [56–167]	147 [96.5–204]	127 [71–199]	0.07
Blood lactate (mmol/L)	2.9 [2–4.3]	2.7 [2.0–3.6]	3 [2.1–5.3]	0.63
Hyperlactatemia (>5.0 mmol/L, %)	17.6	7.4	28.0	0.07
Plasma bicarbonate (mmol/L)	16.2 [13.8–18.4]	14.9 [13.7–16.4]	15.1 [13.7–17.4]	0.08
Metabolic acidosis (<12.2 mmol/L, %)	11.6	11.1	16.0	0.68
Blood glucose (mmol/L)	7.6 [6.3–9.1]	7.9 [6–10.5]	7.5 [6.6–9.1]	0.83
Plasma sodium (mmol/L)	129 [127–132]^b^	132 [130–134]^c^	129 [126–132]	0.007
Plasma creatinine (µmol/L)	26 [21–33]	27 [21–35]	24 [19–32]	0.27
Creatinine clearance (ml/min/1.73 m^2^)	166 [140–199]	158 [123–191]	179 [148–212]	0.16
Renal impairment (<75 ml/min/1.73 m^2^, %)	3.2	3.7	6.0	0.60
Plasma bilirubin (µmol/L)	11.3 [7.2–22]^b^	5.7 [4.1–14]^c^	10.0 [6.5–26.5]	0.002
Hyperbilirubinemia (>35 µmol/L, %)	10.5	0.0	14.6	0.13
Plasma C-reactive protein (mg/dL)	99 [51–154]^b^	41 [16–81]	72 [23–147]	<0.0001
Plasma C-reactive protein>64 mg/L (%)	70.0^b^	37.0	53.2	0.001
Plasma creatine kinase (IU/mL)	67 [17–405]	66 [25–284]	59 [25–592]	0.87
Plasma creatine kinase>2000 IU/mL (%)	9.3	3.8	12.8	0.45
*P. falciparum* density (parasites/µL)	50061 [4817–130344]	0 [0-0]	18715 [155–126894]	0.07#
Hyperparasitemia (>100,000/µL, %)	32.1		32.0	1.0
*P. vivax* density(parasites/µL)	0 [0-0]	1298 [164–4408]	0 [0–107]	<0.0001
South Asian ovalocytosis (SLC4A1Δ27, %)	5.7	3.8	4.3	0.87
Alpha thalassemia (3.7 or 4.2 kb deletions, wt/wt, α^del^/wt, α^del^/α^del^, %)	16.2/38.2/45.6	21.7/34.8/43.5	14.6/36.6/48.8	0.95

Data are, unless otherwise stated, median and [inter-quartile range].

a *P*<0.05 for post-test comparison between *P. falciparum* vs *P. falciparum/vivax*;

b *P*<0.05 for post-test comparison between *P. falciparum* vs *P. vivax*;

c *P*<0.05 for post-test comparison between *P. vivax* vs *P. falciparum/vivax*;

*Kruskal-Wallis or Chi-squared test;

# Mann-Whitney test for *P. falciparum* vs *P. falciparum/vivax*; wt, wildtype; α^del^/wt, heterozygous for either 3.7 or 4.2 kb deletions; α^del^/α^del^, homozygous or compound heterozygous for either 3.7 or 4.2 kb deletion.

Of the sample of 340 children, 19.5%, 5.3% and 5.3%, respectively, had been treated with oral, parenteral or oral plus parenteral antimalarial therapy before admission. Prior treatment was associated with a lower parasite density in children with *P. falciparum* mono-infections (median [IQR] 13,347 [1,784-80,560] vs 68,860 [11,197-171,929] in untreated children, *P*<0.001) but not in children with *P. vivax* mono-infections. For *P. falciparum* mono-infections by nPCR, there was 98.5% concordance with microscopy; two cases were diagnosed as *P. vivax* and two as *P. falciparum/vivax.* For *P. vivax* mono-infections by nPCR, there was 88.9% concordance with microscopy; three cases were diagnosed as *P. falciparum* mono-infections. Six of these seven discordant results were in patients with low parasite densities (<220/µL). For mixed *P. falciparum/vivax* infections, concordance was only 16%, with 68% diagnosed as *P. falciparum* and 16% as *P. vivax* mono-infections by microscopy.

### Presenting features

**Figure 2:**
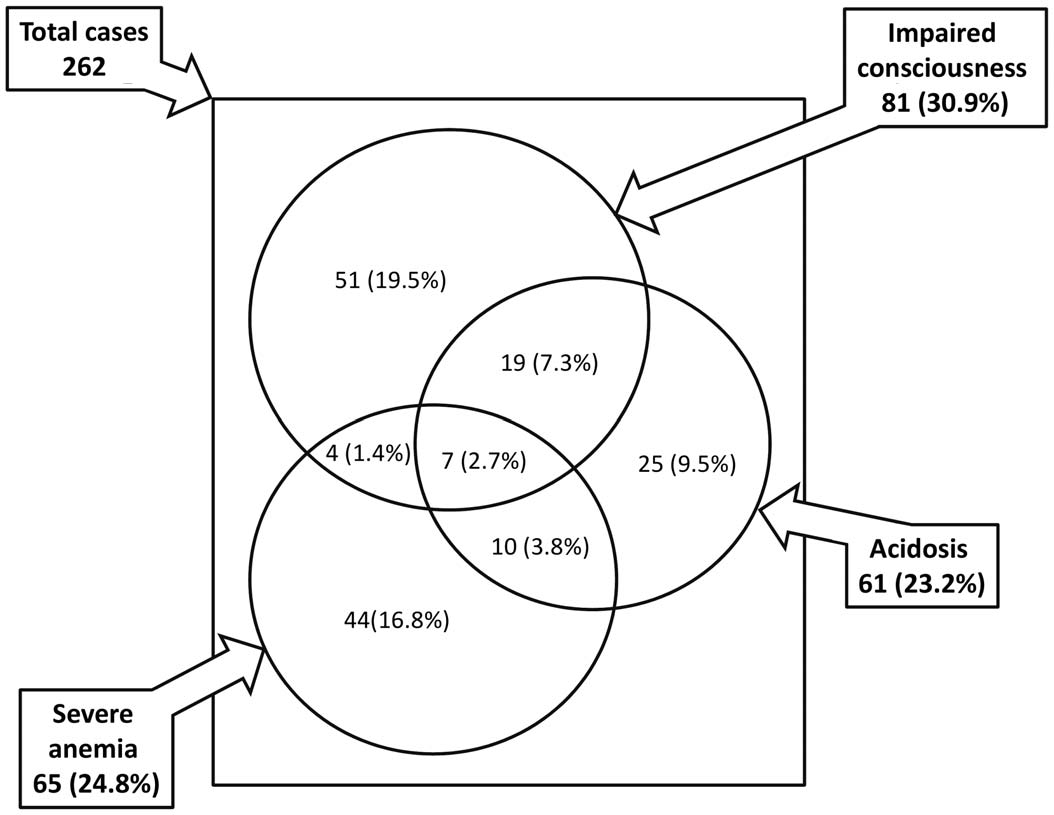
Overlapping clinical phenotypes of severe malaria caused by *Plasmodium falciparum*.

Of 262 children with severe *P. falciparum* mono-infections, 81 (30.9%), 65 (24.8%) and 61 (23.2%) had impaired consciousness, severe anemia, and metabolic acidosis/hyper-lactatemia, respectively (see Figure 2). There were fewer children with the overlap syndrome of impaired consciousness and severe anemia than expected by chance alone (OR and 95% confidence intervals (CI) 0.37 (0.18–0.75), *P* = 0.005) and a greater than expected frequency of impaired consciousness plus acidosis (OR 2.1 (1.1–3.7), *P* = 0.02). Severe anemia plus acidosis occurred at expected frequency (OR 1.2 (0.6–1.9), *P* = 0.61). There were 7 children with all three phenotypes. Only two had hypoglycemia on admission.

Eight children with severe vivax malaria (29.6%) presented with impaired consciousness or deep coma, three (11.1%) with severe anemia, and five (18.5%) with metabolic acidosis or hyperlactatemia. Children with severe vivax malaria had higher median hemoglobin concentrations, smaller spleens, higher median plasma sodium concentrations, and lower median plasma bilirubin and CRP concentrations than children with severe falciparum malaria (see Table 1). After adjustment for the binary variables hyperlactatemia and severe anemia, the proportion of children with respiratory distress was five times that in the severe falciparum malaria group (odds ratio [95% confidence interval] 5.04 [1.84–13.2], *P* = 0.001). There were no other statistically significant differences between the presenting features of severe malaria caused by *P. vivax* or *P. falciparum*.

When compared to those with severe falciparum malaria, children with *P. falciparum/vivax* had a higher median respiratory rate (*P* = 0.014), a lower BCS (*P* = 0.018) and a greater proportion with deep coma (*P* = 0.030; see Table 1). In parallel with the severe *P. falciparum* cases, these children had lower median hemoglobin and serum sodium concentrations, and a higher median plasma bilirubin, than the severe vivax malaria cases. The 5 year-old child with *P. falciparum/malariae* presented with fever, abdominal pain and features of nephrotic syndrome secondary to chronic *P. malariae* infection.

### Clinical course

Most children responded to antimalarial/supportive therapy and were discharged well, including the two hypoglycemic children and the child with *P. falciparum/malariae* malaria. Blood cultures were performed in 258 (75.9%) with contaminants identified in 13 (5.0%). Only two significant isolates were obtained, a non-albicans *Candida* from an admission blood culture from an immuno-competent child and a *Klebsiella pneumoniae* from a child after a week of inpatient treatment for severe malaria. Both children were treated with appropriate antimicrobial therapy and discharged well. A LP was performed in 129 children of whom 11 had>10 white blood cells/µL CSF. Routine bacterial culture, bacterial antigen testing, and India ink staining for *Cryptococcus gattii*, were negative in all cases.

Six children (1.8%) died - one with falciparum (0.4%), one with vivax (3.7%) and four with mixed-species infection (8.0%) - all within 12 hours of admission. The child with falciparum malaria presented deeply comatose and died of cardio-respiratory failure. The fatal *P. vivax* case was a previously well child who presented with breathlessness, and abdominal/lower limb swelling. She had severe respiratory distress, a raised jugular venous pressure and an oxygen saturation of 92% despite supplementary oxygen. Her hemoglobin and blood lactate were 41 g/L and 2.7 mmol/L, respectively. A clinical diagnosis of pericardial effusion with cardiac tamponade was made and she had a cardio-respiratory arrest during attempted pericardiocentesis. Subsequent biochemical analysis revealed renal failure (serum creatinine 460 µmol/L), acidosis and hyperkalemia. The children with mixed *P. falciparum/vivax* infections had the greatest mortality (*P* = 0.003 vs falciparum malaria). Of these four deaths, three were due to cerebral malaria. The fourth child had a hemoglobin concentration of 19 g/L and suffered a cardio-respiratory arrest before blood transfusion could be arranged.

Eight children (2.4%), seven with *P. falciparum* and one with *P. falciparum/vivax*, were discharged with neurologic impairment; three with deep coma on admission developed cortical blindness and five had motor deficits ranging from mild ataxia to spastic quadriparesis. Only two of these eight children had persistent neurologic deficits at follow-up two months later. Another child who recovered from severe vivax malaria with respiratory distress died between discharge and follow-up. When admitted, he had clinical signs of severe pulmonary hypertension and a prior history of echocardiographically confirmed cyanotic heart disease.

The genetic deletion causing SAO was present in 17 of 320 (5.3%) children, including 1, 2 and 14 with severe disease due to *P. vivax*, mixed *P. falciparum/vivax* and *P. falciparum*, respectively. Three children with SAO deletions presented with deep coma (BCS of 1, 2 and 2, respectively) and a further four with impaired consciousness. At least one alpha-thalassemia deletion was observed in 83.6% of the children with severe malaria (see Table 1).

### Population-based rates of severe malaria

Episodes of malaria during a total of 247 person-years of follow-up in the East Sepik cohort study [32] are summarized in Table 2. The data show that the risk of a severe compared with an uncomplicated *P. falciparum/vivax* infection is similar to that with *P. falciparum* alone (3.4% vs 4.2%, *P* = 0.81 by Fisher's exact test) but greater than with *P. vivax* alone (3.4% vs 0.9%, *P* = 0.035). The main manifestations of severe malaria in the 30 children were severe anemia (46.7%) and multiple convulsions (36.7%). Two children with severe falciparum malaria died, one from cardio-respiratory failure secondary to severe anemia (hemoglobin 16 g/L). The other was an anemic child (hemoglobin 63 g/L) with *P. falciparum* whose condition deteriorated after outpatient antimalarial treatment and who died 6 hours later.

**Table 2:** Uncomplicated and severe malaria numbers, and incidence of severe malaria, by infecting *Plasmodium* species from a longitudinal surveillance study of 264 children followed over 17 months in neighbouring East Sepik Province.

*Plasmodium* species	Uncomplicated malaria (n)	Severe malaria (n)	Severe malaria (%)	Incidence of severe malaria per 1,000 person-years at risk
*falciparum*	483	21	4.2†	81.8
*vivax*	461	4	0.9	16.1
*falciparum/vivax*	140	5	3.4†	20.2
Other*	50	0	0	0
Total	1134	30	2.6 (1.8–3.8)¶	121 (82–173)¶

Data are from Lin *et al.* [32].

*Single or mixed infections with *P. malariae* or *P. ovale*;

¶ (95% confidence intervals);

† *P*<0.05 vs *P. vivax*.

## Discussion

The present study confirms that, although severe *P. falciparum* malaria in Melanesian children presents with the same phenotypes as those in African children, the mortality is low (<1%) in a hospital setting. The relatively few cases of severe vivax malaria in our series exhibited presenting features that were similar to those in the children with severe *P. falciparum* malaria but with a higher likelihood of respiratory distress. Severe mixed *P. falciparum/vivax* infections were greater in number than the vivax malaria cases. These children also had comparable admission clinical/laboratory findings to those of the patients with severe falciparum and vivax malaria, but their subsequent mortality was the highest of the three groups. These observations confirm and extend data relating to severe malaria in an area of intense transmission of multiple *Plasmodium* species, especially in relation to mixed-species infections.

Our children with severe falciparum malaria had rates of impaired consciousness, acidosis, anemia, and combinations of these, that were similar to those reported in African studies [1]–[4], [33]–[35]. Impaired consciousness was more often associated with acidosis than severe anemia, again paralleling African data [1]. We had two cases of hypoglycemia, a prevalence of 0.8%. In the recent large-scale African Quinine Artesunate Malaria Trial (AQUAMAT) [36], 10% of children were hypoglycemic at recruitment. This suggests that the nutritional status of our children, including hepatic glycogen reserves (which may reflect differences in duration and/or severity of illness), was better than in African children. Alternatively, a proportion of the patients had received quinine prior to recruitment to AQUAMAT with the risk of attendant hyperinsulinemia [37]. There may also be genetic differences in glucose metabolism between African and Melanesian populations that account for differences in hypoglycemia risk.

Case fatality rates in African children with severe falciparum malaria lie between 3% and 50% [1]–[4]. Our results are consistent with other Melanesian studies in showing lower mortality [5], [6], [18]. This difference could reflect protective genetic factors, relatively infrequent hypoglycemia and bacteremia, and/or better access to quality healthcare. We found alpha-thalassemia deletions in 83.6% of our children, a lower percentage than the 97% found in the general Madang population, and 5.3% had SAO deletions reflecting the results of previous studies in the province [16], [17]. These polymorphisms have previously been shown to be protective against complications of malaria [15]–[17], [38], [39], including near complete protection for SAO against cerebral malaria [16], [38]. Although our study was not designed to assess such a protective effect, the observation that three children with cerebral malaria had the SAO deletion, as did a further four children with impaired consciousness, contrasts with previous studies. This finding suggests that unrecognized favourable genetic traits other than SAO and alpha-thalassemia have emerged through strong selection pressure which might help explain the low mortality observed in our children.

African studies show hypoglycemia-associated mortality of >50% [34], but neither of our two hypoglycemic children died. Similarly, invasive bacterial disease has been detected in 5–8% of African children with severe malaria, especially non-typhoidal *Salmonellae* infection that substantially increase mortality [14], but there was a much lower rate in our cohort. Most of our children had blood cultures performed with an acceptable contamination rate. While we identified invasive bacterial isolates from children severe non-malarial disease (data not shown), only two significant pathogens were obtained from our children with severe malaria. Unregulated antibiotic access is widespread in PNG, but only 8.4% and 7.6%, respectively, had documented amoxicillin or chloramphenicol treatment before admission suggesting that prior antibiotic therapy was not responsible. In addition, no *Salmonellae* were isolated from the present or other children with severe non-malarial disease studied contemporaneously (data not shown). All our children were treated empirically with intramuscular artemether, parenteral chloramphenicol and other supportive therapy [25], and prompt access to good quality standard treatment may also have contributed to their relatively low mortality. Nevertheless, children in the AQUAMAT study had similar management but even those treated with artesunate rather than quinine had a mortality of 8.5% [36].

Severe vivax malaria has been associated with a wide variety of clinical manifestations in adults and children that include severe anemia, altered consciousness, respiratory distress, jaundice, thrombocytopenia, acidosis and renal failure [8], [12], [40]–[44]. There are, however, large between-study differences in the frequency of individual presenting features. This reflects, in part, the fact that few studies have involved prospective collection of clinical and laboratory data sufficient to capture the broad range of features of severe malaria, exclude other causes of severe illness and confirm *Plasmodium* speciation using molecular methods.

A case in point is respiratory distress. In one of two recent prospective studies of severe pediatric *P. vivax* infection from the island of New Guinea [12], children had a limited clinical assessment that included respiratory rate and oxygen saturation. These two features were used to define respiratory distress that was present in approximately 5% of children with severe *P. vivax* malaria. In the second study, which involved prospective community-based morbidity surveillance [41], respiratory distress defined as a respiratory rate>40–50/minute in the presence of chest in-drawing or a history of breathlessness was present in 60.5% of 86 children identified with severe vivax malaria over 8 years. Our prevalence of respiratory distress, based on well-defined specific criteria including a higher respiratory rate threshold, was intermediate between these two figures. Consistent with the other PNG study [41] but not the study from southern Papua [12], respiratory distress was more common in our *P. vivax* cases than those with *P. falciparum*. Acute respiratory distress syndrome (ARDS), interstitial pneumonia and pulmonary oedema can complicate vivax malaria in adults [8]. Since *P. vivax*-infected erythrocytes can bind to cells expressing endothelial receptors known to mediate the cytoadhesion of *P. falciparum* [45], this could reflect parasite microvascular sequestration [46] which may be prominent in the pulmonary capillary bed [47]. This phenomenon may have contributed to respiratory distress in the child with cyanotic heart disease, but there is also evidence that such children may be at increased risk of other manifestations of severe malaria including altered consciousness [48].

There was a single death from *P. vivax* malaria in our cohort. Although this child died during attempted pericardiocentesis, she had severe anemia, respiratory distress, renal impairment and acidosis. In the larger observational study from southern Papua [12] that revealed a case fatality rate for severe vivax malaria in young children (approaching 2%) comparable to that in the present study (3.7%), these presenting features were strongly predictive of death. In a retrospective study from north-eastern Indonesian Papua [11], the overall death rate for severe vivax malaria was 25% in 36 cases. The mortality from severe infections (*P. falciparum* or *vivax*) was lower in children than adults but no pediatric-specific vivax malaria mortality data were reported in this study or a previous prospective observational study from PNG [41].

Our children with severe mixed *P. falciparum/vivax* infections had clinical and laboratory features that were mostly similar to those of the severe falciparum cases despite generally lower *P. falciparum* parasitemias. The exceptions were a higher baseline respiratory rate and a lower BCS. These features suggest that the children in this group were the most severely ill, at risk of the pathophysiologic effects of both parasite species with the possibility that *P. vivax* microvascular cytoadherence as observed in lung [47] and spleen [46] might also occur in the brain.

Other studies have found that, in contrast to the present data, severe anemia is less common in severe *P. falciparum/vivax* infections than severe falciparum or vivax malaria [12], that mixed infections are more likely than *P. vivax* infections to be associated with respiratory distress [41], and that coma is least likely in mixed infections [41]. These apparent inconsistencies may reflect between-study differences in patient selection, definitions of complications, and the availability and quality of clinical/laboratory data including accurate microscopy and/or molecular confirmation of *Plasmodium* species. Nevertheless, our data indicate that WHO criteria for severe malaria [13] that are based on studies of *P. falciparum* encompass the spectrum of severe infections caused by *P. vivax* with or without co-incident *P. falciparum*.

Although from a study with a different design, the community-based longitudinal data from a cohort of similarly aged East Sepik children who had similar red-cell polymorphisms and who were from an area of comparable malaria transmission [32], suggest that both *P. falciparum* mono-infections and *P. falciparum/vivax* infections in a PNG child are more likely to progress to severe disease than *P. vivax* mono-infections. Indeed, the numbers of severe cases in these categories (21, 5 and 4, respectively) were consistent those of the present study (262, 50 and 27), suggesting that our hospitalized patients were representative of malaria in the community. The mortality in our series was greatest in severe *P. falciparum/vivax* infections, consistent with the presenting clinical and laboratory features of these children. In the two other studies with mortality data [11], [12], there was no significant difference between mixed and single-species infections but no pediatric-specific outcomes were included. Thus, our data are the first to suggest that mixed-species infections carry an adverse prognosis in children.

Our study had limitations. It is possible that the most severely ill children in the study catchment area die before they can be transferred for inpatient care, thus compromising the representative nature of our sample. Due mainly to issues with obtaining a sufficient volume of blood from a small, distressed and severely ill child, we could not exclude bacteremia in every case, while diagnostic tests for viral and fungal pathogens were not possible in this clinical setting. Obtaining *post mortem* tissue samples that may have clarified underlying pathology is culturally problematic in PNG.

The strengths of the present study were its prospective standardized data collection, the use of nPCR as a way of compensating for the known limitations of microscopy, and the availability of bacteriology facilities that enabled detection of common invasive bacterial infections. Such a rigorous approach has been used in few previous descriptive studies of severe childhood malaria. Our data confirm the lack of sensitivity of microscopy in identifying mixed species infections [49], [50], with low-level *P. vivax* densities that can be missed by microscopy in this situation probably reflecting density dependent cross-species regulation [51]. Although nPCR is not a quantitative test and the relative contribution of each species to the overall parasitemia remains uncertain, microscopic misdiagnosis of mixed infections in previous studies may have obscured the association with more severe disease observed in the present patients. Given the presenting features and clinical course of our patients, it is unlikely that there were significant numbers of cases with diagnoses that would have been identified by specialized serologic and other tests. Indeed, in the case of encephalitis, more than one third of cases remain undiagnosed in a resource-rich healthcare setting [52]. It has been suggested that parasite density thresholds are employed in areas where asymptomatic parasitemia is common in otherwise healthy children [53], [54]. While the severe malaria-attributable fraction or diagnostic specificity increases with higher parasitemia, sensitivity is reduced. The choice of threshold will depend on factors such as local transmission, age-dependent malarial immunity and genetic factors modulating infection severity.

The present study of well-characterized children from an area of intense transmission of multiple *Plasmodium* species has identified significant differences between the presentation and outcome of severe *P. falciparum*, *vivax* and *falciparum/vivax* infections in PNG. The low mortality observed for severe falciparum malaria does not appear related to high endemicity of *P. vivax* but may reflect protective genetic factors, better prior nutrition, and/or less exposure/susceptibility to secondary bacterial infection than in African children. Severe vivax malaria presents with features seen in severe *P. falciparum* infections but respiratory distress is more prominent, suggesting preferential cytoadherence of this parasite within the lung microvasculature. Severe *P. falciparum/vivax* infections in PNG children appear to have the worst prognosis, perhaps because they share the adverse presenting features of the respective mono-infections. These findings provide impetus for further research on the pathogenic potential of *P. vivax* infections when this parasite is present alone and especially in combination with other *Plasmodium* species.

## References

[1] Marsh K, Forster D, Waruiru C, Mwangi I, Winstanley M (1995). Indicators of life-threatening malaria in African children.. N Engl J Med.

[2] Mockenhaupt FP, Ehrhardt S, Burkhardt J, Bosomtwe SY, Laryea S (2004). Manifestation and outcome of severe malaria in children in northern Ghana.. Am J Trop Med Hyg.

[3] Schapira A, Solomon T, Julien M, Macome A, Parmar N (1993). Comparison of intramuscular and intravenous quinine for the treatment of severe and complicated malaria in children.. Trans R Soc Trop Med Hyg.

[4] Imbert P, Gerardin P, Rogier C, Ka AS, Jouvencel P (2002). Severe falciparum malaria in children: a comparative study of 1990 and 2000 WHO criteria for clinical presentation, prognosis and intensive care in Dakar, Senegal.. Trans R Soc Trop Med Hyg.

[5] Allen SJ, O'Donnell A, Alexander ND, Clegg JB (1996). Severe malaria in children in Papua New Guinea.. QJM.

[6] Maitland K, Williams TN, Peto TE, Day KP, Clegg JB (1997). Absence of malaria-specific mortality in children in an area of hyperendemic malaria.. Trans R Soc Trop Med Hyg.

[7] Stace J, Bilton P, Coates K, Stace N (1982). Cerebral malaria in children: a retrospective study of admissions to Madang Hospital, 1980.. P N G Med J.

[8] Price RN, Douglas NM, Anstey NM (2009). New developments in *Plasmodium vivax* malaria: severe disease and the rise of chloroquine resistance.. Curr Opin Infect Dis.

[9] Luxemburger C, Ricci F, Nosten F, Raimond D, Bathet S (1997). The epidemiology of severe malaria in an area of low transmission in Thailand.. Trans R Soc Trop Med Hyg.

[10] Price RN, Simpson JA, Nosten F, Luxemburger C, Hkirjaroen L (2001). Factors contributing to anemia after uncomplicated falciparum malaria.. Am J Trop Med Hyg.

[11] Barcus MJ, Basri H, Picarima H, Manyakori C, Sekartuti (2007). Demographic risk factors for severe and fatal vivax and falciparum malaria among hospital admissions in northeastern Indonesian Papua.. Am J Trop Med Hyg.

[12] Tjitra E, Anstey NM, Sugiarto P, Warikar N, Kenangalem E (2008). Multidrug-resistant *Plasmodium vivax* associated with severe and fatal malaria: a prospective study in Papua, Indonesia.. PLoS Med.

[13] WHO (2000). Severe falciparum malaria.. Trans R Soc Trop Med Hyg.

[14] Berkley J, Mwarumba S, Bramham K, Lowe B, Marsh K (1999). Bacteraemia complicating severe malaria in children.. Trans R Soc Trop Med Hyg.

[15] Yenchitsomanus PT, Summers KM, Bhatia KK, Cattani J, Board PG (1985). Extremely high frequencies of alpha-globin gene deletion in Madang and on Kar Kar Island, Papua New Guinea.. Am J Hum Genet.

[16] Allen SJ, O'Donnell A, Alexander ND, Mgone CS, Peto TE (1999). Prevention of cerebral malaria in children in Papua New Guinea by southeast Asian ovalocytosis band 3.. Am J Trop Med Hyg.

[17] Allen SJ, O'Donnell A, Alexander ND, Alpers MP, Peto TE (1997). alpha+-Thalassemia protects children against disease caused by other infections as well as malaria.. Proc Natl Acad Sci U S A.

[18] Karunajeewa HA, Reeder J, Lorry K, Dabod E, Hamzah J (2006). Artesunate suppositories versus intramuscular artemether for treatment of severe malaria in children in Papua New Guinea.. Antimicrob Agents Chemother.

[19] Michon P, Cole-Tobian JL, Dabod E, Schoepflin S, Igu J (2007). The risk of malarial infections and disease in Papua New Guinean children.. Am J Trop Med Hyg.

[20] Smith T, Hii JL, Genton B, Muller I, Booth M (2001). Associations of peak shifts in age–prevalence for human malarias with bednet coverage.. Trans R Soc Trop Med Hyg.

[21] Manning L, Laman M, Townsend MA, Chubb SP, Siba PM (2011). Reference intervals for common laboratory tests in melanesian children.. Am J Trop Med Hyg.

[22] UNAIDS (2010). Global Report Fact Sheet - Oceania.. http://www.unaids.org/en/media/unaids/contentassets/documents/factsheet2010/20101123_FS_oceania_em_en.pdf.

[23] Molyneux ME, Taylor TE, Wirima JJ, Borgstein A (1989). Clinical features and prognostic indicators in paediatric cerebral malaria: a study of 131 comatose Malawian children.. Q J Med.

[24] Laman M, Manning L, Hwaiwhange I, Vince J, Aipit S (2010). Lumbar puncture in children from an area of malaria endemicity who present with a febrile seizure.. Clin Infect Dis.

[25] Paediatrics Society of PNG (2005). Standard treatment for common illnesses of children in PNG..

[26] Karunajeewa HA, Mueller I, Senn M, Lin E, Law I (2008). A trial of combination antimalarial therapies in children from Papua New Guinea.. N Engl J Med.

[27] Moosa AA, Quortum HA, Ibrahim MD (1995). Rapid diagnosis of bacterial meningitis with reagent strips.. Lancet.

[28] Snounou G, Viriyakosol S, Jarra W, Thaithong S, Brown KN (1993). Identification of the four human malaria parasite species in field samples by the polymerase chain reaction and detection of a high prevalence of mixed infections.. Mol Biochem Parasitol.

[29] Imrie H, Fowkes FJ, Michon P, Tavul L, Hume JC (2006). Haptoglobin levels are associated with haptoglobin genotype and alpha+ -thalassemia in a malaria-endemic area.. Am J Trop Med Hyg.

[30] Jarolim P, Palek J, Amato D, Hassan K, Sapak P (1991). Deletion in erythrocyte band 3 gene in malaria-resistant Southeast Asian ovalocytosis.. Proc Natl Acad Sci U S A.

[31] Schwartz GJ, Haycock GB, Edelmann CM, Spitzer A (1976). A simple estimate of glomerular filtration rate in children derived from body length and plasma creatinine.. Pediatrics.

[32] Lin E, Kiniboro B, Gray L, Dobbie S, Robinson L (2010). Differential patterns of infection and disease with *P. falciparum* and *P. vivax* in young Papua New Guinean children.. PLoS One.

[33] Dzeing-Ella A, Nze Obiang PC, Tchoua R, Planche T, Mboza B (2005). Severe falciparum malaria in Gabonese children: clinical and laboratory features.. Malar J.

[34] White NJ, Waller D, Crawley J, Nosten F, Chapman D (1992). Comparison of artemether and chloroquine for severe malaria in Gambian children.. Lancet.

[35] Ranque S, Poudiougou B, Traore A, Keita M, Oumar AA (2008). Life-threatening malaria in African children: a prospective study in a mesoendemic urban setting.. Pediatr Infect Dis J.

[36] Dondorp AM, Fanello CI, Hendriksen IC, Gomes E, Seni A (2010). Artesunate versus quinine in the treatment of severe falciparum malaria in African children (AQUAMAT): an open-label, randomised trial.. Lancet.

[37] Davis TM (1997). Antimalarial drugs and glucose metabolism.. Br J Clin Pharmacol.

[38] Genton B, al-Yaman F, Mgone CS, Alexander N, Paniu MM (1995). Ovalocytosis and cerebral malaria.. Nature.

[39] Mockenhaupt FP, Ehrhardt S, Gellert S, Otchwemah RN, Dietz E (2004). Alpha(+)-thalassemia protects African children from severe malaria.. Blood.

[40] Lacerda MV, Alexandre MA, Santos PD, Arcanjo AR, Alecrim WD (2004). Idiopathic thrombocytopenic purpura due to vivax malaria in the Brazilian Amazon.. Acta Trop.

[41] Genton B, D'Acremont V, Rare L, Baea K, Reeder JC (2008). *Plasmodium vivax* and mixed infections are associated with severe malaria in children: a prospective cohort study from Papua New Guinea.. PLoS Med.

[42] Kochar DK, Das A, Kochar SK, Saxena V, Sirohi P (2009). Severe *Plasmodium vivax* malaria: a report on serial cases from Bikaner in northwestern India.. Am J Trop Med Hyg.

[43] Alexandre MA, Ferreira CO, Siqueira AM, Magalhaes BL, Mourao MP (2010). Severe *Plasmodium vivax* malaria, Brazilian Amazon.. Emerg Infect Dis.

[44] Kochar DK, Tanwar GS, Khatri PC, Kochar SK, Sengar GS (2010). Clinical features of children hospitalized with malaria - a study from Bikaner, northwest India.. Am J Trop Med Hyg.

[45] Carvalho BO, Lopes SC, Nogueira PA, Orlandi PP, Bargieri DY (2010). On the cytoadhesion of *Plasmodium vivax*-infected erythrocytes.. J Infect Dis.

[46] Fernandez-Becerra C, Yamamoto MM, Vencio RZ, Lacerda M, Rosanas-Urgell A (2009). *Plasmodium vivax* and the importance of the subtelomeric multigene vir superfamily.. Trends Parasitol.

[47] Anstey NM, Handojo T, Pain MC, Kenangalem E, Tjitra E (2007). Lung injury in vivax malaria: pathophysiological evidence for pulmonary vascular sequestration and posttreatment alveolar-capillary inflammation.. J Infect Dis.

[48] Okeniyi J, Kuti B (2008). Cerebral malaria in children with cyanotic heart diseases: the need for a closer look.. Congenit Heart Dis.

[49] Ohrt C, Purnomo, Sutamihardja MA, Tang D, Kain KC (2002). Impact of microscopy error on estimates of protective efficacy in malaria-prevention trials.. J Infect Dis.

[50] Zimmerman PA, Mehlotra RK, Kasehagen LJ, Kazura JW (2004). Why do we need to know more about mixed *Plasmodium* species infections in humans?. Trends Parasitol.

[51] Bruce MC, Donnelly CA, Alpers MP, Galinski MR, Barnwell JW (2000). Cross-species interactions between malaria parasites in humans.. Science.

[52] Granerod J, Ambrose HE, Davies NW, Clewley JP, Walsh AL (2010). Causes of encephalitis and differences in their clinical presentations in England: a multicentre, population-based prospective study.. Lancet Infect Dis.

[53] Bejon P, Berkley JA, Mwangi T, Ogada E, Mwangi I (2007). Defining childhood severe falciparum malaria for intervention studies.. PLoS Med.

[54] Schellenberg JR, Smith T, Alonso PL, Hayes RJ (1994). What is clinical malaria? Finding case definitions for field research in highly endemic areas.. Parasitol Today.

